# Association between Multi-Dose Drug Dispensing and Quality of Drug Treatment – A Register-Based Study

**DOI:** 10.1371/journal.pone.0026574

**Published:** 2011-10-31

**Authors:** Christina Sjöberg, Christina Edward, Johan Fastbom, Kristina Johnell, Sten Landahl, Kristina Narbro, Susanna Maria Wallerstedt

**Affiliations:** 1 Department of Geriatrics, Sahlgrenska University Hospital/Mölndal, Mölndal, Sweden; 2 Pharmaceutical Unit, Department of Health, County Council of Kalmar, Kalmar, Sweden; 3 Aging Research Center, Karolinska Institutet and Stockholm University, Stockholm, Sweden; 4 Department of Public Health and Community Medicine, Geriatrics Unit, University of Gothenburg, Sahlgrenska University Hospital/Mölndal, Mölndal, Sweden; 5 Administrative Department of Health, Regional Secretariat, Region Västra Götaland, Göteborg, Sweden; 6 Department of Clinical Pharmacology, Sahlgrenska University Hospital, Göteborg, Sweden; University of Modena and Reggio Emilia, Italy

## Abstract

**Background:**

In the elderly in Scandinavia, multi-dose drug dispensing (MDD) is a common alternative to ordinary prescriptions (OP). MDD patients receive their drugs in unit bags, one for each dose occasion. The prescribing procedure differs between MDD and OP. The aim of the present study was to investigate the association between MDD and quality of drug treatment (QDT).

**Methodology/Principal Findings:**

A cross-sectional study was performed of all inhabitants in Region Västra Götaland alive on December 31st 2007, aged ≥65 years, with ≥1 prescribed drug and ≥2 health care visits for ≥2 diagnoses for obstructive pulmonary disease, diabetes mellitus, and/or cardiovascular disease in 2005–2007 (n = 24,146). For each patient, drug treatment on December 31st 2007 was estimated from drugs registered in the Swedish Prescribed Drug Register. QDT was evaluated according to established quality indicators (*≥10 drugs*, *Long-acting benzodiazepines*, *Drugs with anticholinergic action*, *≥3 psychotropics*, and *Drugs combinations that should be avoided*). Logistic regression, with adjustments for age, sex, burden of disease, and residence, was performed to investigate the association between MDD and QDT. Mean age was 77 years, 51% were females, and 20% used MDD. For all quality indicators, the proportion of patients with poor QDT was greater in patients with MDD than in patients with OP (all P<0.0001). Unadjusted and adjusted odds ratios (95% confidence intervals) for poor QDT (MDD patients vs. OP patients) ranged from 1.47 (1.30–1.65) to 7.08 (6.30–7.96) and from 1.36 (1.18–1.57) to 5.48 (4.76–6.30), respectively.

**Conclusions/Significance:**

Patients with MDD have poorer QDT than patients with OP. This cannot be explained by differences in age, sex, burden of disease, or residence. These findings must be taken into account when designing alternative prescribing systems. Further research is needed to evaluate causative factors and if the findings also apply to other dose dispensing systems.

## Introduction

Dose dispensing systems are widespread over the world, but limited knowledge is available on safety aspects of such systems [1]. An important safety concern is quality of prescribing. Indeed, the prescriber rather than the nursing and pharmacy services accounts for the majority of medication errors, as well as for the majority of severe medication errors [2]. Quality of prescribing can be measured by drug-specific quality indicators. These are quantitative measures based on international literature on quality of drug use. In Sweden, the National Board of Health and Welfare has developed quality indicators to measure quality of drug treatment in older people [3]. These have been used both in research [4], [5] and for benchmarking [6].

In Sweden, about 182,000 out of 9 million inhabitants use the multi-dose drug dispensing (MDD) system *ApoDos*®, which is intended for patients on regular medication with difficulties in handling their own drugs due to impaired physical or cognitive function. MDD is used by community-dwelling patients as well as those who live in nursing homes. In the Region Västra Götaland, where this study was performed, eleven per cent of people 65 years or older use the MDD system as opposed to ordinary prescriptions (OP). In people 75 years or older the corresponding figure is 19%. In the MDD system, drugs that should be ingested concomitantly are delivered in machine-dispensed unit bags. The multi-dose unit bags are labelled with patient data, drug contents, date, and time for intake. The bags are usually delivered every fortnight, but deliveries within a couple of days can be made upon request. Drugs that cannot be dispensed into the unit bags, for instance liquids as well as effervescent or chewing tablets, are delivered in ordinary labelled original packages. These drugs comprise about half of all drugs prescribed via the MDD system [7]. A specific multi-dose drug prescription is required, where all concomitant drugs can be viewed. This is not possible with OP. In both systems, written and electronic prescriptions can be used. Within the MDD system, drugs for one year utilization can be prescribed at a time. The same applies to OP, but these have to be filled four times a year. There are other differences in the prescribing procedure between the MDD system and OP, and apprehensions have been raised that these may affect prescribing habits [7], [8].

A previous study based on drug register data has reported an association between MDD and poor quality of drug treatment [9]. However, that study did not take into account diagnoses or residence. These two covariates should be of importance for drug treatment, since diagnoses make up the basis for drug treatment, and MDD is almost mandatory for patients staying in nursing homes in our region. Therefore, we wanted to make a more profound investigation with inclusion of these important factors. Thus, the aim of the present study was to investigate the association between MDD and quality of drug treatment, with adjustments for age, sex, burden of disease, and residence.

## Materials and Methods

### Participants

The study cohort encompassed all inhabitants in Region Västra Götaland alive at December 31^st^ 2007 who met all of the inclusion criteria: (i) ≥65 years, (ii) ≥1 dispensed drug registered in the Swedish Prescribed Drug Register at any time from the start of the register (July 1^st^ 2005) to December 31^st^ 2007, and (iii) ≥2 health care visits for ≥2 diagnoses within the International Statistical Classification of Diseases and Related Health Problems (ICD10) codes for obstructive pulmonary disease (J44–J46), diabetes mellitus (E10–E14), or cardiovascular disease (I10–I13, I20–I25, I50) registered in the regional health care consumption database (VEGA) in 2005–2007. The latter restriction of the study population was made to make the comparison groups more alike regarding burden of disease.

The study complies with the Declaration of Helsinki. Ethics approval was obtained from the Regional Ethical Review Board in Gothenburg (Dnr 358-08). Informed consent was not applicable since data were obtained and analysed anonymously. According to Swedish regulations ethics approval must precede extraction of register data.

### Description of procedures

The study cohort was extracted by linkage of individual data from the Swedish Prescribed Drug Register with data from the VEGA database by the unique personal identity number. The Swedish Prescribed Drug Register contains data on all prescribed drugs that are dispensed to a specific individual at Swedish pharmacies. The VEGA database contains consumption of health care for all inhabitants of Region Västra Götaland, including in-hospital as well as primary care diagnoses.

For patients with OP, a medication list at December 31^st^, 2007 was estimated from drugs registered in the Swedish Prescribed Drug Register during the three month period preceding this date (i.e. October 1^st^–December 31^st^, 2007) [4]. The rational for this time frame was the Swedish regulations, where a maximum of three months' drug use is reimbursed at one purchase occasion. The date of the filling of the prescription, the amount of drug dispensed, and the prescribed dosage was used to estimate the duration of the dispensed volume of a drug [4], and if this covered treatment at December 31^st^. When prescribed dosage was incomplete or missing, a daily dose for the actual drug was looked up from a table derived from the same dataset, with mean daily doses from prescriptions with known dosage information. For drugs prescribed as needed we assumed a dosage of 50% of that for regular drugs. Moreover, we assumed a daily dose of 1 defined daily dose (DDD) [10] for drugs for external use and for the eye.

In the MDD system, drugs are either dispensed in unit bags with prescriptions filled every fortnight, or delivered in original packages. In the Swedish Prescribed Drug Register, prescribed dosages are currently not included for patients with MDD. Therefore, we assumed dose-dispensed drugs to be current medications if filled within 14 days before December 31^st^ 2007, whereas the use of drugs delivered in whole packages was assessed using the method described above for OP with incomplete or missing dosage information.

For each patient, quality of drug treatment was assessed by five drug-specific quality indicators, developed by the Swedish National Board of Health and Welfare: *Ten or more drugs*, *Long-acting benzodiazepines*, *Drugs with anticholinergic effects*, *Three or more psychotropics*, and *Drug combinations that should be avoided*. These indicators are all inverted, that is, presence of such treatment, regularly or as needed, indicates poor quality of drug treatment. The quality indicators are described in Table 1.

**Table 1:** Description of drug-specific quality indicators used in the present study.

Indicator	Included drugs	ATC-code^1^
Ten or more drugs	all drugs	
Long-acting benzodiazepines	diazepam	N05BA01
	nitrazepam	N05CD02
	flunitrazepam	N05CD03
Drugs with anticholinergic effects	(anticholinergic) drugs for functional gastrointestinal disorders	A03AB, A03BA, A03BB
	(anticholinergic) antiemetics	A04AD
	antiarrythmics class Ia	C01BA
	urinary antispasmodics	G04BD
	opioids in combination with antispasmodics	N02AG
	anticholinergic (anti-Parkinson drugs)	N04A
	low potency antipsychotics	N05AA, N05AB04, N05AF03
	hydroxyzine	N05BB01
	non-selective monoamine reuptake inhibitors (antidepressants)	N06AA
	antihistamins	R05CA10, R06AA02, R06AB, R06AD, R06AX02
Three or more psychotropics	antipsychotics	N05A
	anxiolytics	N05B
	hypnotics and sedatives	N05C
	antidepressants	N06A
Drug combinations that should be avoided	drugs with D-interactions, as defined in the Pharmaceutical Specialities in Sweden (FASS) [17]	

1 ATC-code, Anatomical Therapeutical Chemical classification code [10].

Patient diagnoses were extracted from the VEGA database. As an estimate of burden of disease, the number of different diagnoses (ICD10-code, 3 digits) in hospital and primary care was summarized for each patient. Furthermore, all patients were categorized as either with or without a psychiatric diagnosis within the ICD10-codes covering dementia, organic brain disorders, psychotic disorders, abuse, affective disorders, anxiety, and sleep disorders (F00–F03, G30, G31.8A, F06, F09, F1–F4, F51.0, F51.9, G47.0, G47.9).

Residence (nursing home or community-dwelling) on October 1^st^ 2007 was extracted from the Swedish Social Service Register.

### Statistical analysis

The Student's t-test and the Chi-square test were used for comparisons between patients with and without MDD. Logistic regression was performed to evaluate the association between MDD and the quality of drug treatment. The results were adjusted for age, sex, burden of disease, and residence. Since psychiatric diseases may be associated with both residence and MDD, and several of the drug-specific quality indicators involve drugs intended for psychiatric diseases, a sensitivity analysis was performed with *Any psychiatric diagnosis* as a dummy variable included in the model. Collinearity between variables in the model was investigated with Pearson's correlation. The statistical analyses were performed by SPSS 17.0.

## Results

A total of 24,146 patients were included (mean age [standard deviation]: 77 [7.2] years; 51% female); 4,927 (20%) with MDD and 19,219 (80%) with OP. Characteristics of patients are presented in Table 2. Compared with patients with OP, patients with MDD were older and more often female, had more drugs and diagnoses, and more often lived in nursing homes (all P<0.0001).

**Table 2:** Characteristics of patients.

		MDD	OP	P-value
		n = 4,927	n = 19,219	
Age, years, mean ± SD		81.2±7.2	75.5±6.7	<0.0001
Female sex, n (%)		2,860 (58.0)	9,411 (49.0)	<0.0001
Number of drugs at December 31^st^ 2007, mean ± SD		10.3±3.9	6.6±3.6	<0.0001
Diagnoses, mean ± SD	Total	17.2±7.6	12.7±6.7	<0.0001
	Obstructive pulmonary disease	1,732 (35.2)	6,560 (34.1)	0.18
	Diabetes mellitus	3,844 (78.0)	14,634 (76.1)	0.0056
	Cardiovascular disease	4,875 (98.9)	18,992 (98.8)	0.46
	Psychiatric disease	1,623 (32.9)	1,654 (8.6)	<0.0001
Living in nursing homes, n (%)		1,362 (27.6)	113 (0.6)	<0.0001

MDD, multi-dose drug dispensing; OP, ordinary prescriptions; SD, standard deviation.

The proportion of patients with poor quality in drug treatment according to the quality indicators varied between 5.9% and 55% for patients with MDD, and between 2.6% and 19% for patients with OP (Table 3). Patients with MDD showed poorer quality on all quality indicators than patients with OP (all P<0.0001).

**Table 3:** Number of patients with poor quality in prescribing according to drug-specific quality indicators.

	MDD	OP	P-value
	n = 4,927	n = 19,219	
Ten or more drugs, n (%)	2,717 (55.1)	3,671 (19.1)	<0.0001
Long-acting benzodiazepines, n (%)	292 (5.9)	619 (3.2)	<0.0001
Drugs with anticholinergic effects, n (%)	630 (12.8)	1,035 (5.4)	<0.0001
Three or more psychotropics, n (%)	792 (16.1)	506 (2.6)	<0.0001
Drug combinations that should be avoided (D-interactions), n (%)	401 (8.1)	1,094 (5.7)	<0.0001

MDD, multi-dose drug dispensing; OP, ordinary prescriptions.

The unadjusted odds for a patient to have poor quality in drug treatment according to the five drug-specific quality indicators were between 1.47 and 7.08 times higher for patients with MDD (Table 4). After adjustments for age, sex, burden of disease, and residence, the odds were between 1.36 and 5.48; the greatest odds were found for quality indicators on number of concomitant drugs. For all quality indicators, the odds for poor quality in drug treatment were greater for MDD than for the other variables included in the model, and in three out of five quality indicators, the confidence intervals between MDD and the other variables did not overlap.

**Table 4:** Unadjusted and adjusted odds ratios for poor drug treatment according to the five drug-specific quality indicators.

	Ten or more drugs	Long-acting benzodiazepines	Drugs with anticholinergic effects	Three or more psychotropics	Drug combinations that should be avoided (D-interactions)
Multi-dose drug dispensing^1^, OR (95% CI)	5.21 (4.87–5.57)	1.89 (1.64–2.18)	2.58 (2.32–2.86)	7.08 (6.30–7.96)	1.47 (1.30–1.65)
Multi-dose drug dispensing^1^, AOR (95% CI)	3.88 (3.58–4.21)	1.61 (1.36–1.91)	2.32 (2.05–2.63)	5.48 (4.76–6.30)	1.36 (1.18–1.57)
Age^2^, AOR (95% CI)	0.99 (0.98–0.99)	0.99 (0.98–1.00)	0.97 (0.96–0.98)	0.96 (0.95–0.97)	0.99 (0.98–0.99)
Female sex^3^, AOR (95% CI)	1.27 (1.20–1.36)	1.48 (1.29–1.70)	1.33 (1.20–1.48)	1.42 (1.26–1.60)	1.14 (1.02–1.27)
Number of diagnoses^2^, AOR (95% CI)	1.09 (1.09–1.10)	1.05 (1.04–1.05)	1.04 (1.03–1.05)	1.05 (1.04–1.06)	1.04 (1.03–1.04)
Nursing home^4^, AOR (95% CI)	1.35 (1.19–1.53)	0.83 (0.63–1.09)	1.24 (1.03–1.49)	2.20 (1.87–2.60)	0.89 (0.71–1.12)

OR, odds ratio; CI, confidence interval; AOR, adjusted odds ratio.

1 Ref: ordinary prescriptions;

2 continuous;

3 Ref: male sex;

4 Ref: community-dwelling.

When the results were also adjusted for *Any psychiatric diagnosis*, the odds ratio (95% confidence interval) for poor quality in drug treatment was; use of: *Ten or more drugs*, 3.85 (3.54–4.18); *Long-acting benzodiazepines*, 1.39 (1.17–1.65); *Drugs with anticholinergic effects*, 2.15 (1.89–2.44); *Three or more psychotropics*, 4.01 (3.48–4.64); and *Drug combinations that should be avoided* (D interactions), 1.37 (1.19–1.58).

Correlation coefficients between MDD and the other variables in the model were 0.32 (age), 0.07 (sex), 0.26 (number of diagnoses), 0.46 (residence), and 0.24 (any psychiatric diagnosis).

## Discussion

### Principal findings

Our results indicate that MDD is negatively associated with quality of drug treatment. Up to five times as many patients with MDD had poor quality of drug treatment according to drug-specific quality indicators. Interestingly, this finding can neither be explained by their being more ill nor their need to stay in a nursing home, since both number of different diagnoses and residence were included in the model. Indeed, the odds ratios for poor quality of drug treatment for MDD were high as compared with other patient characteristics. Thus, the MDD system seems to be a prominent determinant for poor quality of drug treatment. This finding is interesting, since it indicates that a technology (MDD) which aims to solve a problem (to facilitate and increase safety in drug handling for the patient and the health care staff) may introduce new problems (poorer quality in drug treatment), as previously discussed [11].

The greatest differences between patients with and without MDD were found for quality indicators concerning number of drugs, *Ten or more drugs* and *Three or more psychotropics*. These results could not be explained by a greater burden of disease for patients with MDD. The results confirm previous assumptions that the MDD system increases the number of drugs [8], and thus adjustments for number of drugs, as made in a previous study [9], may diminish the estimates of the effects of MDD on drug treatment. Even after adjustment for psychiatric disease, four times as many patients with MDD had poor quality according to the quality indicator *Three or more psychotropics*. One may speculate that the different prescribing procedures involved in MDD and OP may affect the quality of prescribing. In the MDD system, all prescriptions can easily be renewed at the same time, which could lead to less frequent withdrawals of drugs. In OP, on the other hand, all prescriptions need to be renewed one at a time. To the best of our knowledge, no scientific literature is available on the effects of different prescribing procedures on inclination to make changes in drug treatment, that is, additions, withdrawals, or dosage adjustments, over time.

When a patient is treated with numerous drugs, the risk of *Drug combinations that should be avoided* would be expected to increase [12]. In the present study, the risk for potentially serious drug-drug interactions was increased in patients with MDD but to a lower degree than could be expected from their use of many drugs. One explanation for this may be that drug-drug interaction warnings based on the complete medication list of the patient are given in the MDD prescribing procedure. When prescribing to patients with OP, drug-drug interaction warnings only occur for drugs prescribed concomitantly, that is, the complete medication list is unavailable. Interestingly, previous results concerning MDD patients and *Drug combinations that should be avoided* are somewhat contradictory; the proportion of patients with such combinations was greater for patients with MDD than for patients with OP (8.8% *vs.* 3.7%), but after adjustments for number of dispensed drugs, the odds including confidence interval was <1.0 [9].

### Limitations

The present study has several limitations. First, the cross-sectional study design does not allow conclusions concerning causality between MDD and poor quality in drug treatment. Thus, we cannot rule out if MDD leads to low quality of drug treatment, or if low quality of drug treatment leads to MDD. Further longitudinal research is needed to clarify causality. Moreover, patients with and without MDD are obviously not alike. Other factors not included in the multivariate model may be of importance. However, we have tried to enhance precision and make the study more efficient by restricting the study population, that is, to only include older people with established obstructive pulmonary disease, diabetes mellitus, and/or cardiovascular disease. Thus, the study population is a subset of all people ≥65 years in the Region Västra Götaland (24,146 out of 265,819 [9.1%] at December 31^st^ 2007 [13]). Furthermore, we have made an additional attempt to control for confounders by including important covariates in the model, such as burden of disease, psychiatric disease, and residence.

Second, our analysis is based on register data only, and the estimated medication list may not reflect the true drug use, that is, drug use may be both over- and underestimated. Moreover, drugs are dispensed more frequently for patients with than without MDD. This may make the estimated medication list of a patient with MDD more accurate than that of a patient with OP. It cannot be ruled out that the differing registration frequency may have affected the results of the present study. However, the principle of estimating actual drug use based on prescriptions filled during a three months period has been employed in several previous studies [14], [15], [16], and indeed, the present method used for estimation of a medication list is the one used by the National Board of Health and Welfare for calculating quality indicators [6].

Third, the drug-specific quality indicators employed in the present study do not provide all aspects of quality of drug treatment. Indeed, all the quality indicators in our study reflect inappropriate drug use. The study does not examine undertreatment with drugs, for example, when treatment with bisphosphonates is lacking in patients at high risk of fractures.

Fourth, data from the Swedish Prescribed Drug Register do not include drugs for in-hospital use or drugs sold over-the-counter. Moreover, the register is incomplete as regards drugs used in nursing homes; occasionally in such residencies, medications from drug storerooms are dispensed without being registered in the Swedish Prescribed Drug Register, e.g. antibiotics for short-time use and temporary analgesics.

### Future research

Summarized, patients with MDD have poorer quality in their drug treatment than patients with OP, and this cannot be explained by differences in age, sex, burden of disease, or residence. These findings should have implications for countries which already have or plan to introduce dose dispensing systems. Further research is needed to evaluate causative factors and if the findings also apply to other dose dispensing systems.
